# Discrepancies between empirical and theoretical probability in human binary choices within the game of Go

**DOI:** 10.3389/fpsyg.2026.1594220

**Published:** 2026-04-30

**Authors:** Yong-Hwan Kim, Soo Hyun Jeong, Soo-Chan Kim, Yongkuk Kim, Kwon-Seok Chae

**Affiliations:** 1 Neuroscience Program, School of Allied Health Sciences, Boise State University, Boise, ID, United States; 2 Department of Baduk Studies, Myongji University, Yongin, Republic of Korea; 3 School of Electronic and Electrical Engineering, Institute of IT Convergence, Hankyong National University, Anseong, Republic of Korea; 4 Department of Mathematics, Kyungpook National University, Daegu, Republic of Korea; 5 Department of Biology Education, Kyungpook National University, Daegu, Republic of Korea; 6 Brain Science and Engineering Institute, Kyungpook National University, Daegu, Republic of Korea

**Keywords:** decision-making, empirical probability, Go game, Go players, stone selection, zero-sum binary choice

## Abstract

To make a decision or determine the order of things, people commonly use binary choices such as tossing a coin, with an assumption that the empirical probability equals to the theoretical probability. However, recent understanding on empirical coin-toss probability is contrasting, i.e., even or uneven by quantic or deterministic mechanism, wherein such discrepancies yet remain to be resolved. Here, we investigated whether the probability of stone selection in the game of Go is equal for both parties across Go games to establish the stone selection as a binary choice model for studying the discrepancy between theoretical and empirical probability. The stone selection process is considered as a sequential strategic interaction similar to other binary choice games such as matching pennies. The large-scale data analyses of professional Go matches (*n* = 21,212 among 311 Go masters) showed that the black stone selection was significantly higher for higher-ranked players compared with lower-ranked players. Moreover, *in situ* stone selection games between amateur Go players (the number of participants: *n* = 293, 2 games each) demonstrated that the amateur players with upper master levels had significantly higher chances for selecting black stones than players with lower master levels. The results support that actual chance for human binary choice may not be equal to theoretical odds, unlike usual expectations. The present study may contribute to providing input or insights to expand its discussion regarding the discrepancy between theoretical and empirical probability in binary choices, including strategy in game theory and elucidating the underlying mechanisms.

## Introduction

To decide the order for taking a turn in games or sports, people often toss a coin as a binary choice (McGarry and Franks, 2000; Hahn and Warren, 2009; Piper et al., 2019), assuming the even probability of the choices for both sides (McGarry and Franks, 2000; Hahn and Warren, 2009). The outcome of probabilistic binary choices between two parties is implemented in numerous zero-sum games including poker, chess, and Go, wherein players’ choices are frequently mutually exclusive and can be linked to competitive rewards (Nash, 1951; Koller and Megiddo, 1992). In this study, we assessed the stone selection process in Go games as an example of binary choice. The Go game (called “*baduk*” in Korean) is one of the oldest board games that is mainly popular in East Asian countries (Davies, 1977; Chaslot et al., 2006). As an intellectual strategy game, Go has been the most challenging board game for artificial intelligence to defeat professional human players until the recent feat by the computer program AlphaGo (Benson, 1976; Silver et al., 2016).

Since a Go match starts with the first move by a black stone, a stone selection should be conducted before a Go match begins, which is a typical process for deciding who takes the black stone (Kim and Kim, 1995). At the beginning of a match, two players sit facing each other across the board and perform the stone-selection process. In the first step, the player 1 who has longer professional career than the other player (player 2), grabs a handful of white stones in his/her fist and then places the fist on the board to hide the stones. The number of white stones is usually greater than 10. In the second step, the player 2 places one or two black stone(s) on the board to determine who would take black or white stones for the match. The player 1 reveals whether the number of the white stones is odd or even by aligning the white stones as separate sets comprised of two or four stones. If both white and black stones turned out to be matched either odd or even, each player keeps the stone color (s)he already has. However, if the two sets of stones were mismatched: odd–even or even–odd, players trade the stone buckets.

This process is important for Go players as it affects the actual winning probability of individual players with black or white stones and both players’ game strategies to win (Kim and Kim, 1995; Kim and Jeong, 2005; Kim, 2007). The majority of Go players favor playing black to take advantage of moving first as a whole, even though individual differences in playing styles may incline to prefer to play black or white. To reduce the advantage of playing black, the compensation points (i.e., Komi) in Korean Go rules have largely been increased starting from 5.5 up to 7.5 over time. Nonetheless, the stone selection process is believed to be a fair binary choice because both players participate in the two decision steps, and the probability for each stone is considered 0.5. However, it is unclear whether the empirical probability is truly equal to the theoretical probability in binary choices including the stone selection, as people easily assume. For instance, the scientific understanding of coin-toss probability is limited, and earlier studies stand in a sharp contrast to the belief either the deterministic model to be unequal (Diaconis et al., 2007; Clark and Westerberg, 2009; Ferrie and Combes, 2014; Bartoš et al., 2023) or a quantum phenomenon with theoretically even odds (Adler, 2014; Albrecht and Phillips, 2014). Yet, the stone selection in Go game is a double-binary choice process, whereas coin-toss can be a single binary choice between two parties. The stone selection process in Go game can be analyzed as a sequential strategic interaction similar to other binary choice games, such as matching pennies and rock-paper-scissors (Glimcher, 2001; Semmann et al., 2003). In game theory terms, this represents a zero-sum game with incomplete information, where players must make sequential decisions under the uncertainties (von Neumann and Morgenstern, 1944; Nash, 1951). In this regard, the stone selection process is a binary choice game involving two players, each of whom chooses a strategy from two options, either intentional or random choice, at the decision steps.

Recently, we reported that humans can exploit geomagnetic sensing for probabilistic abstract decision-making in stone selection of Go games (Oh et al., 2025). One of the rationales for the study was that human magnetic sense might be implicated in discrepancies between theoretical and empirical probabilities in the binary choices under certain circumstances (Chae et al., 2023). However, the empirical discrepancies in the study were not convincingly demonstrated, and the biological meaning of the discrepancies was little addressed. As a follow-up and revisiting study (Chae et al., 2023; Oh et al., 2025), we assessed whether there are discrepancies between theoretical and empirical probabilities in the stone selection of Go games by statistical analyses and explored the potential biological insights of the results. In this study, two main questions were tackled to elucidate the followings: (1) Correlation between “black stone (%)” and “player ranking” in the stone selection data from 18 years of professional Go matches, (2) Correlation between “black stone (%)” and “level (Dan)” in the *in situ* stone selection games data from 16 locations of Go classrooms or training academies.

## Methods

### Stone selections in professional Go matches

**Figure 1:**
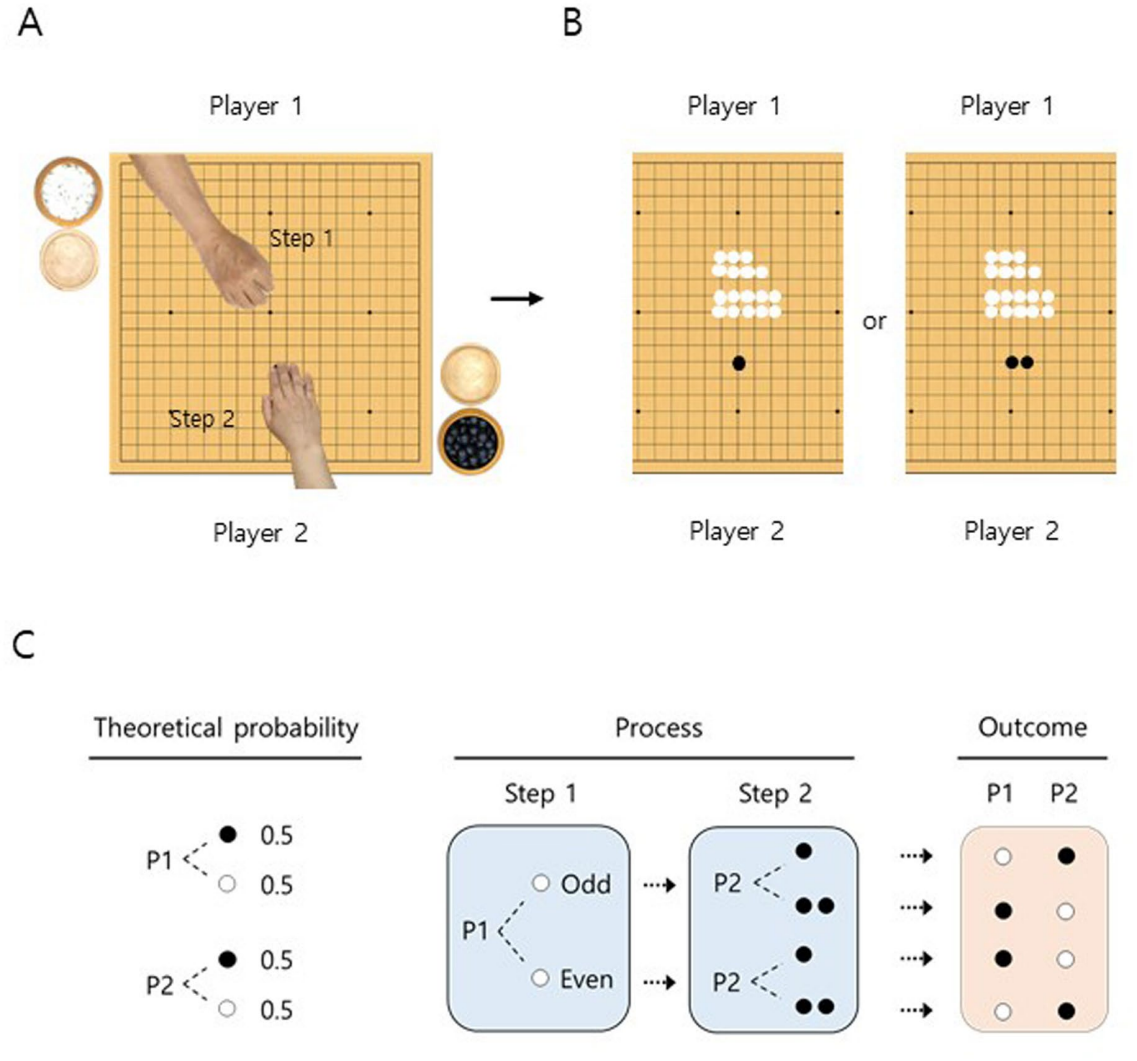
Equal theoretical probability of stone selection in Go games. A schematic drawing of the stone selection process. **(A)** In the first step, player 1 grabbed a handful of white stones and placed them on the board while keeping the stones in the fist. In the second step, player 2 placed one or two black stone(s) on the board. **(B)** Player 1 revealed whether the number of white stones (17 in this example) was odd or even. **(C)** The theoretical probability for both players to choose either the black or the white stone is equal. In the flow-chart for the process and outcome of stone selection, dashed lines or arrows indicate the possible routes for the probable cases in stone selection. If the number of the white and black stone(s) on the board is matched to be either odd or even, players keep the same color of the stones next to them. If not, they switch stone colors for the match. P1, player 1; P2, player 2; white circles, white stones; black circles, black stones.

All the final-round professional Go matches were held in either a quiet room or a ballroom. The stone selection was conducted according to the rules of Korea Baduk Association (2023) as described in the introduction (see Figures 1A–C and Supplementary Movie S1, Youtube, 2023).

### The analysis of stone selection data from professional Go matches

**Figure 2:**
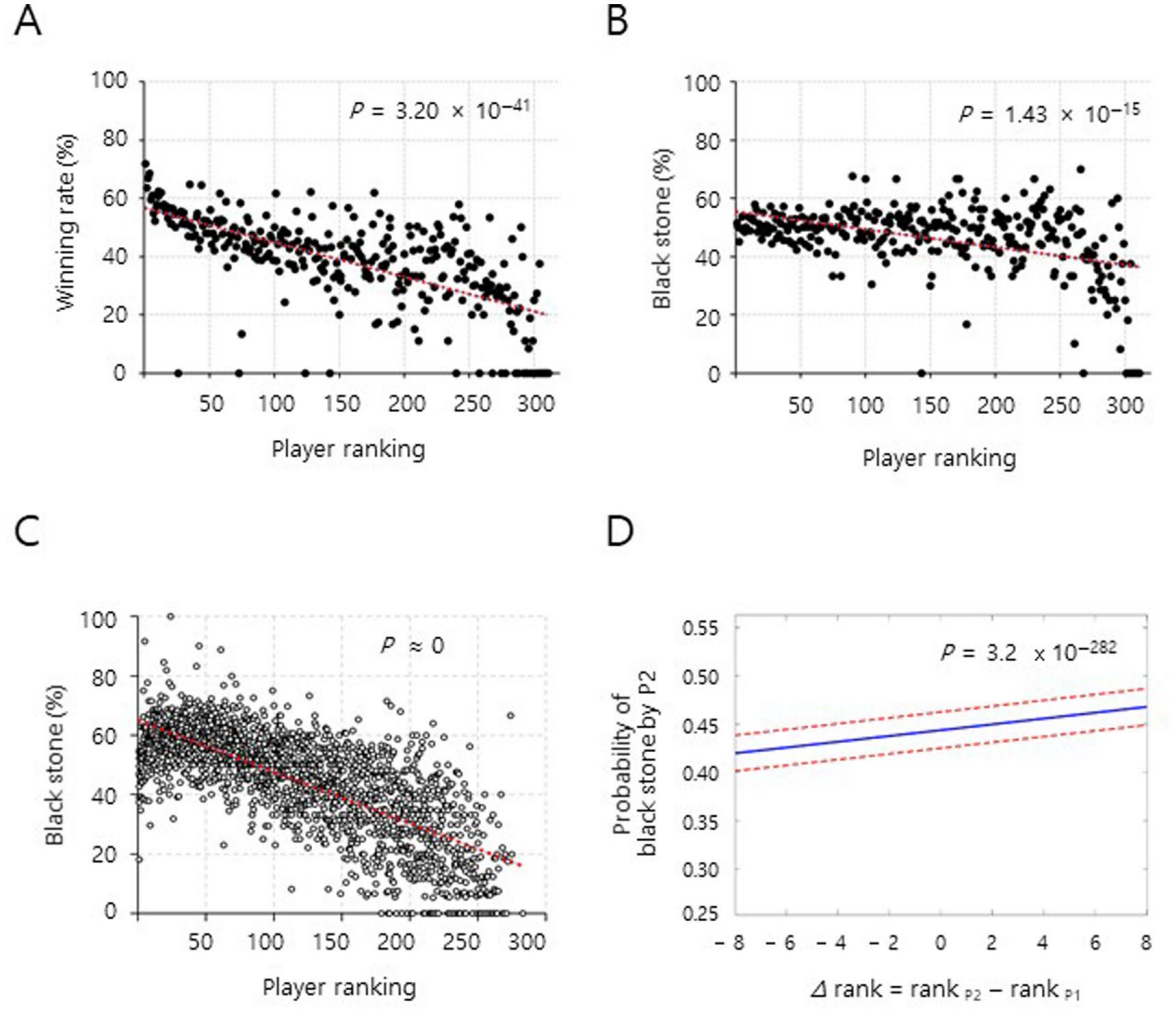
Discrepancies between the empirical and theoretical probability of stone selection. The winning rate and the black stone rate were analyzed and displayed based on 311 players listed in the player ranking as of June 2018. Linear fit analyses **(A–C)** and BT–GLMM analysis **(D)** of the data from the final rounds of 21,212 Go matches among 311 players over 18 years are displayed. **(A)** A significant inverse correlation between players’ ranking and winning rates was detected. *y* = − 0.12 *x* + 56.60, *p* = 3.20 × 10^−41^ (except outliers (*n* = 24, 7.7%) who had 0% winning rate due to the lack of winning in final-round matches, *p* = 5.47 × 10^−38^), Pearson’s *r* = − 0.67, *n* = 311. **(B)** A significant inverse correlation between players’ ranking and black stone (%) was found. *y* = − 0.061 *x* + 55.51, *p* = 1.43 × 10^−15^ (except outliers (*n* = 11, 3.5%) with a 0% black stone (%) due to having no black stone selection in any final round matches, *p* = 9.38 × 10^−10^), Pearson’s *r* = − 0.43, *n* = 311. (**C**) A significant inverse correlation between players’ ranking and year-based black stone (%) from 2009 to 2017, wherein players who had less than eight matches in the corresponding year were not included. *y* = − 0.17 *x* + 64.96, *P* ≈ 0, Pearson’s *r* = − 0.73, *n* = 1,861. (**D**) Predicted probability that player 2 selects black stones as a function of rank difference (*∆*rank = rank _P2_ − rank _P1_) under the BT–GLMM. P1, player 1; P2, player 2; solid line, estimated mean; dashed lines, 95% CI. See the details in the Supplementary Table S3.

Data from professional Go matches supervised by the Korea Baduk Association were obtained through the official request by the corresponding author. Data elements include the date and location of matches, the title of the championship, the status of the matches (e.g., preliminary/final round or special match), stone colors for players, the names of winners/losers, and the compensation points given to the white stone player. The locations were at many different places in the country, despite some matches were held outside of the country (e.g., China or Japan). A substantial portion of preliminary-round matches with insufficient information and some final-round matches between players who were unaffiliated with the Korea Baduk Association, were not included in the dataset. Thus, the analyzed dataset includes 21,212 matches out of 73,730 total matches (28.8%) held between 2000 and 2017 (Supplementary Table S1). The analyzed data include 311 professional Go players (262 men; 49 women) listed in the player ranking, issued as of July 2018, which was the very first ranking after February 2018 (the end of the 2017 Go tournament season). In a separate analysis, the dataset comprised the player ranking as of July 2018 and the black stone (%) for each player in every year between 2009 and 2017; players who had fewer than eight matches (approximately one-third of the average number of matches per player annually) in the corresponding year were excluded. Supplementary analyses examining ranking data from 2009 to 2018 indicate high temporal stability at both the individual and structural levels, supporting the use of the 2018 ranking as a reference for long-term player strength. A specific number for the data used in each analysis was indicated in the text, figure legends for Figures 2, 4 or Supplementary Tables S2–S5.

**Figure 3:**
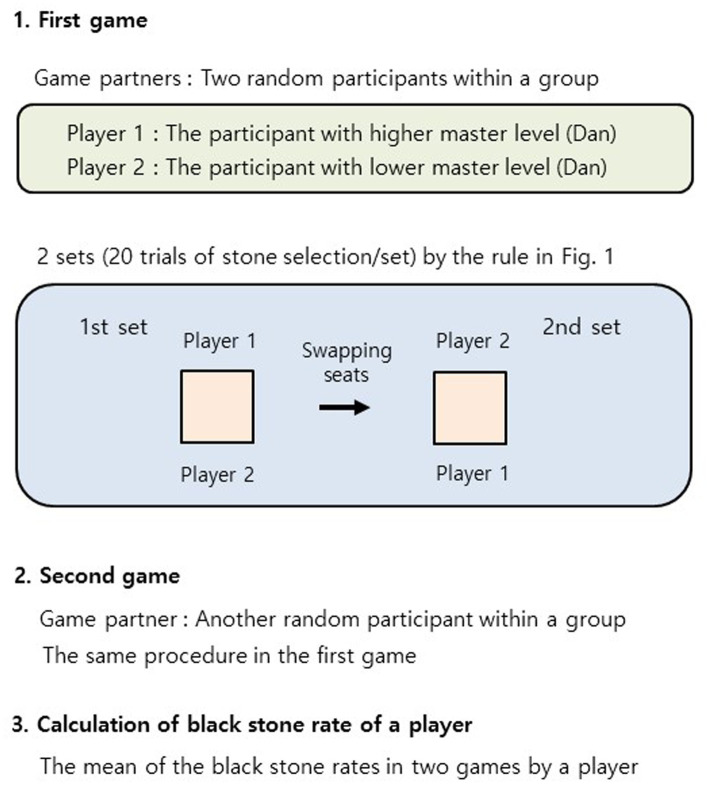
A descriptive diagram of *in situ* stone selection games. The procedures of *in situ* stone selection games are listed in a step-by-step manner. In cases both players are in the same level (Dan) for the game, an elderly participant was the player 1.

**Figure 4:**
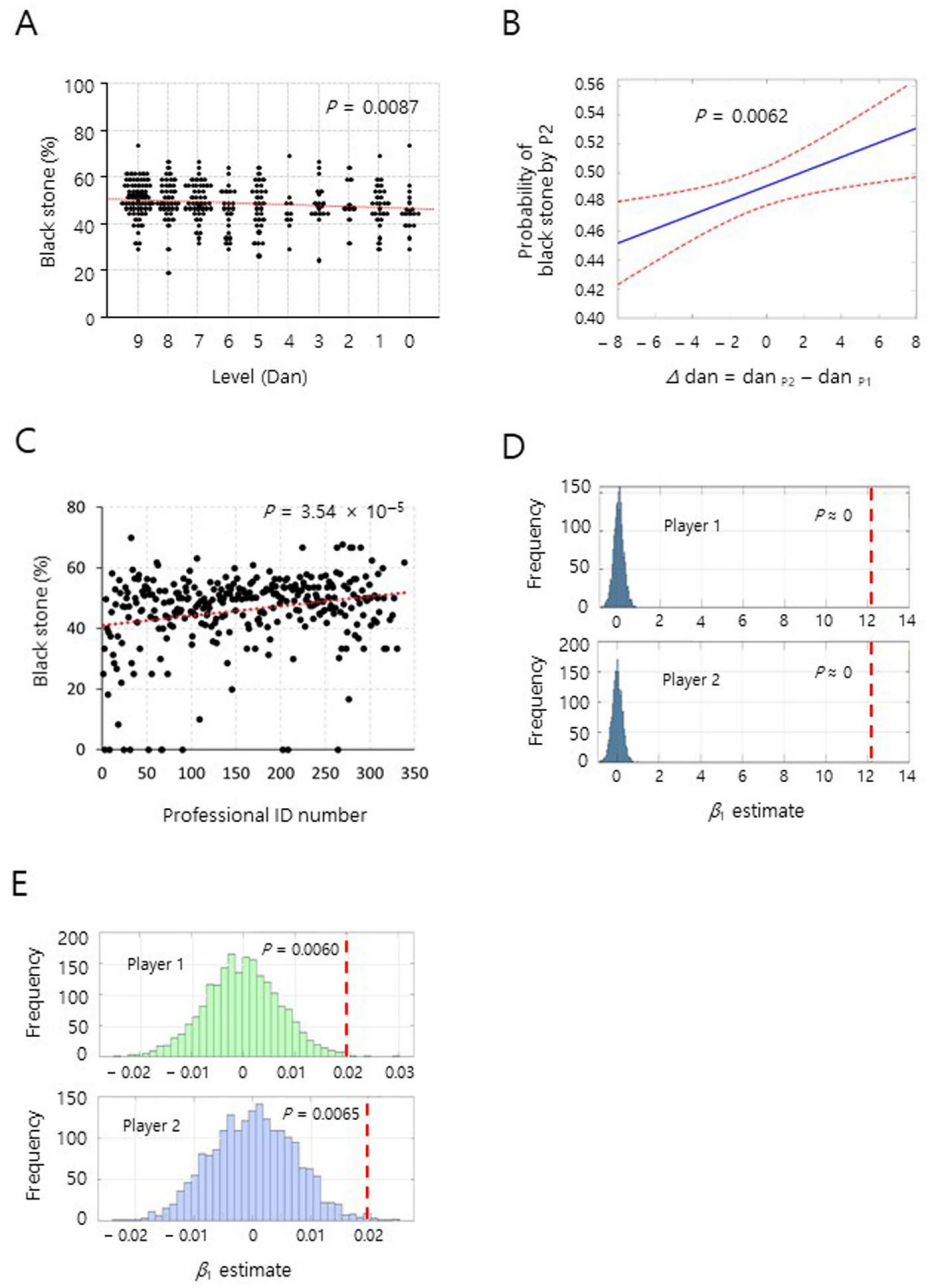
Unequal empirical probability of *in situ* stone selection games. Linear fit analyses **(A,C)**, the BT–GLMM analysis **(B)**, and null simulation analyses **(D,E)** were performed. **(A)** The analysis of *in situ* stone selection games among 293 amateur players showed a significant correlation between master level (Dan) and black stone (%). *y* = 0.483 *x* + 47.10, *p* = 0.0087, Pearson’s *r* = 0.15, *n* = 293. Higher Dan numbers indicate higher master levels. **(B)** Predicted probability that player 2 selects black stones as a function of Dan (level) difference (*∆*dan = dan _P2_ − dan _P1_) under the BT–GLMM. P1, player 1; P2, player 2; solid line, estimated mean; dashed lines, 95% CI. See the details in the Supplementary Table S3. **(C)** A significant correlation between player ID numbers and black stone (%) was confirmed. *y* = 0.032 *x* + 40.94, *p* = 3.54 × 10^−5^, Pearson’s *r* = 0.23, *n* = 311. A player ID number is a unique number given to a person when (s)he becomes a professional Go player. Higher numbers indicate relatively shorter professional career. Null distributions of *β*_1_ estimate for player 1 and player 2 **(D,E)** are displayed under the random black/white stone assignment [Bernoulli (0.5)]. Each of the dashed lines indicates the observed coefficient from actual raw data that are the same as used in Figures 2B, 4A **(A)**. See the details in the Supplementary Table S5.

### *In situ* stone selection games

*In situ* stone selection games were executed at 16 different locations distributed over the country in either quiet classrooms (in the Department of Baduk Studies at Myongji University or the Korea Baduk middle and high school) or training rooms (at private amateur Go training academies) between November 2018 and September 2020. These were performed in the cooperation with the institutions at the appointed time under the supervision of an experimenter—volunteered 293 participants (220 men; 73 women) of 16 groups (18.3 ± 7.4 people/group, mean ± SD). Before the beginning of games, all the participants were informed of the aim and procedure of the games, the financial compensation for the participation, and the additional reward for the selection of black stone. These stone selection games were conducted by the same rule as the stone selection in professional Go matches, with some exceptions. (1) Two participants in each group were randomly chosen for the first game. In the first step, a participant with higher master level (“Dan,” 0–9 grade) was player 1, and the other participant with lower level was player 2 in the match. In cases of the same Dan for the participants, the elderly participant would be the player 1. (2) The match comprised two sets and 20 trials of stone selection per set. Using the same game rules, both players took a turn to grab white stones in the trials in a set—player 1 grabbed white stones in the first trial, and player 2 grabbed white stones in the second trial for the stone selection (see Supplementary Data S1 for record form). All the participants were instructed to grab at least 10 white stones in the first step (if it was less than 10, the trial was nullified and retried) and not to use a predetermined pattern but to decide the number of black stones (one or two) extemporaneously in the second step during the entire game. The game information was determined and then recorded on a record form for each trial, set, and game by the participants themselves, with both players’ confirmation under the supervision of an experimenter (the correction rate of judgment error in the game information, approximately 2%). (3) The players switched seats, “player 1” and “player 2” in a swap, before starting the second set in a game (Figure 1A); the same game rule and procedure in (2) were applied to the second set. (4) The black stone (%) of a player in a game was calculated as the number of black stone selection out of total number of trials (i.e., 40) × 100. (5) All the participants played the second game with another random opponent player within a group, and a player’s black stone (%) in the analyzed data was the mean of the two games of a player.

### Statistical analysis

To determine the significance of data, a linear fit analysis and the Bradley-Terry generalized linear mixed model (BT–GLMM) were applied to the stone selection data from professional Go matches and *in situ* stone selection games. The linear fit analyses were performed using the software Origin 2019 (OriginLab, Northampton, USA). *p*-value < 0.05 was regarded as significant. To account for the pairwise nature of the data (Bradley and Terry, 1952), the BT-GLMM was applied using the MatLab (MathWorks, Natick, USA). Importantly, the BT–GLMM used in this study does not model match outcomes or winning probabilities, as in conventional Bradley–Terry models. Instead, the dependent variable represents stone selection behavior (Black choice). We do not estimate player-specific fixed ability parameters {*β_i_*} as typically analyzed in the Bradley–Terry ability model; pairwise heterogeneity is captured through a match-level random effect, which can be re-expressed as *β_i_* − *β_j_* under i.i.d. Gaussian player effects. Rank difference (Δrank) is included directly as a fixed effect, while event- and match-level random effects are specified as random intercepts to account for clustering and pairwise dependence, as is standard in mixed-effects logistic regression for clustered binary outcomes (Wang and Louis, 2003). In particular, if


$$\beta_{i}\overset{i.i.d}{\sim }N(0,{\sigma^{2}}_{\text{player}})$$

then the match-level term can be written as


$$u_{\text{match}}=\beta_{i}-\beta_{j}∼N(0,2{\sigma^{2}}_{\text{player}})$$

Simple logistic regression would underestimate standard errors and yield invalid inference because matches within the same event and between the same players are not independent. The BT–GLMM includes event-level and match-level random intercepts, which appropriately account for clustering, repeated participation, and the dependence inherent to paired-comparison data. We index each game by an ordered player pair (*i*, *j*), where *i* denotes player 1 and *j* denotes player 2 in the recorded matchup. Each observation corresponds to one game. Let Y*_ij_* ∈ {0, 1} be the match-level response, where Y*_ij_* = 1 if player *j* chooses Black (and Y*_ij_* = 0 otherwise), and define *p_ij_* = Pr(Y*_ij_* = 1). Define Δrank*_ij_* = rank*_j_* − rank*_i_* (Dan difference), and Δgender*_ij_* analogously when included. Let *t* denote the event (tournament) identifier, and let *m*(*i*, *j*) denote the pairing identifier (i.e., repeated encounters between the same two players share the same pairing ID), which is used as a random intercept to capture within-pair dependence and game-to-game variability. The BT–GLMM is specified as

$$\text{logit}(p_{\mathrm{ij}})=\beta_{0}+\beta_{1}\;\text{Δran}k_{\mathrm{ij}}+\beta_{2}\;\text{Δgende}r_{\mathrm{ij}}+u_{t}+u_{m(i,j)},$$

where *u_t_* ~ *N*(0, σ^2^_event_) and *u*_*m*(*i*, *j*)_ ~ *N*(0, σ^2^_match_). This is a standard mixed-effects logistic regression specification for clustered binary outcomes (Wang and Louis, 2003). Here, *p_ij_* represents the model-implied (conditional) probability that player *j* chooses Black in the matchup (*i*, *j*), given the covariates and random effects. This formulation allows us to (1) model the competitive pair structures directly; (2) quantify the population-level tendency while accounting for random variation across events. In the analyzed data, the gender of participants was somewhat imbalanced: 262 men vs. 49 women in professional Go matches and 220 men vs. 73 women in *in situ* stone selection games, which may cause the coefficient estimate to be volatile and/or biased. However, these gender ratios from professional Go matches (15.8%) or *in situ* stone selection games (24.9%) are within the normal range of professional female Go players in Korea (21.7%), based on the 2023 survey (Kang et al., 2024). To justify the inclusion of event-level random effects, we conducted restricted likelihood ratio tests (RLRTs) separately for the professional and amateur datasets, reflecting the distinct nature of events (official tournaments vs. classrooms or training rooms). Specifically, we compared a full model including an event-level random intercept with a reduced model excluding it, using the test statistic RLRT = −2 (reduced−full). The event-level random effect was significant for professional matches but not for amateur matches (Supplementary Table S2); therefore, the final models include an event-level random intercept for professionals but exclude it for amateurs. *p* < 0.05 was regarded as significant. For null simulation analysis using the same BT–GLMM framework, we generated null distributions for each of the players under a random-choice scenario, in which each player’s stone selection was replaced by a random binary variable [Bernoulli (0.5)] (Pan and Lin, 2005). This procedure was repeated 2000 times, yielding a null distribution centered around zero. The coefficient of *β*_1_ from the simulated data was then compared to that from the real data. The *p*-value of less than 0.05 was considered as significant.

## Results

### Equal theoretical probability of stone selection in the game of Go

To accommodate the rules for a stone selection, procedural descriptions are illustrated visually in Figures 1A–C and Supplementary Movie S1. It is assumed that both players have the equal chance of 0.5 to select black or white stones because they are engaged in the two-step decision process (double-binary choice) without knowing their stone color, until the number of white stones is revealed to be odd or even.

### Discrepancies between the empirical and theoretical probabilities of stone selection

In order to examine if the empirical probability equals to the theoretical probability, we initially collected large-scale stone selection data from professional matches between January 2000 and February 2018 supervised by Korea Baduk Association (2023), an organization that oversees professional Go games in South Korea (Paradigm 1, introduction). We analyzed 21,212 Go final-round matches held during the aforementioned period (Supplementary Table S1), with 311 players listed in the player ranking as of June 2018 (see Methods). Notably, final-round matches are more important and competitive than preliminary matches for professionals, because of the prize money and enhancement of ranking. As expected, the winning rate was associated with higher ranking for all players by linear fit analysis (Figure 2A and Supplementary Data S2). However, contrary to the expectation that the black (and white) stone rate would be about 50%, irrespective of player ranking, there was a significant positive correlation between the black stone selection rate and higher player ranking in the entire data set of the period aforementioned (Figure 2B and Supplementary Data S3) and a set of year-based data between 2009 and 2017 (Figure 2C and Supplementary Data S4). Note that the black stone selection rate of a player is considered to be ‘black stone (%)’. Given the pairwise competitive nature of the stone selection (Kim and Kim, 1995; Kim and Jeong, 2005), the BT-GLMM was applied to confirm the unexpected results. The estimated coefficient for *∆*rank (*β*_1_) was positive and statistically significant, indicating that the probability of selecting black stones increases as ranking differences are higher (Figure 2D and Supplementary Table S3). Restricted likelihood ratio tests showed that the event-level random effect was appropriately considered in the BT-GLMM for professional matches, whereas the same effect was excluded from the BT-GLMM for the *in situ* stone selection games below (Supplementary Table S2). Noticeably, the black stone selection rate was marginally affected by gender (Supplementary Table S4). These results provide compelling evidence that the black stone selection rate of upper-ranked players was significantly higher than that of lower-ranked players in professional Go matches.

### Unequal empirical probability of *in situ* stone selection games

To ascertain the unequal empirical probability in the stone selection, we conducted stone selection games *in situ* by the same rule above between amateur Go players at special Go schools or Go training academies where they attended for years to become professional Go players (Paradigm 2 in introduction; Figure 3, see Methods). Our results from the linear fit analysis and BT-GLMM also support that amateur Go players with upper master levels had significantly higher actual chances for selecting black stones than players with lower master levels (Figures 4A,B, Supplementary Table S3, and Supplementary Data S5). Note that the black stone selection rate was little affected by gender, similarly in professionals (Supplementary Table S4). All the Go professionals have extensive experience that presumably affects the results in Figures 2B–D (Hahn and Warren, 2009). However, against a potential assumption that the black stone (%) could be relatively higher in players with longer professional career (largely older Go player), the black stone (%) was significantly higher in players with relatively shorter professional career by linear fit analysis (Figure 4C and Supplementary Data S6). Note that the higher professional ID numbers indicate shorter professional careers. This unexpected result suggests that higher chances of choosing the black stone was somewhat inversely correlated with the length of professional Go career. The results above in Figures 2, 4 imply that the unequal empirical probabilities in the stone selections may reflect strategic behavior rather than random choice in the stone selections. To examine the premise, we conducted null simulation analyses using the same raw data from professionals and amateurs in Figures 2B, 4A, respectively. The observed *β*_1_ from the real data fell well outside of the 95% quantile of the null distributions for both player 1 and 2, in both professional and amateur matches, indicating that the significant differences in black stone acquisition cannot be attributed to random chance (Figures 4D,E and Supplementary Table S5). This result supports that the players’ stone selections are non-random and strategy-dependent, consistent with the hypothesis that players respond to their opponents when they select stones. Taken together, the results in Figures 2, 4, and Supplementary Table S2–S5 show that the empirical probabilities of binary stone selection in Go games were noticeably unequal to the theoretical probability of 0.5.

## Discussion

The initiative of this study is to determine whether the odds to choose a head or a tail in splitting a chance such as flipping a coin, would be even between two parties. Our unexpected findings strongly suggest that theoretically equal probability in a binary choice was not observed in stone selection for the game of Go. Although we usually assume that it is a randomized chance, our observation suggests that higher ranking professional Go players had a higher chance to grab black stones, which was favorably correlated to winning a match (Kim and Kim, 1995; Kim and Jeong, 2005). Notably, given the big data from quasi-random professional matches among hundreds of Go masters during a long period, the unequal empirical probability found in this study may not be merely due to a coincidence or random chances. The significance of this inequality is strengthened by the fact that players’ prevailing competitive preference for the black stone is well recognized irrespective of player ranking (Kim and Kim, 1995; Kim and Jeong, 2005; Kim, 2007). Moreover, the discrepancy between the theoretical and empirical probabilities was further supported by the large-scale empirical data from the *bona fide in situ* stone selection games between amateur Go players at different locations. These results suggest the potential implication of unknown factors to contribute to the unequal probability for the selection of black stone under the reward-motivated circumstances.

In the present study, we could not provide direct evidence for the underlying mechanism in which higher-ranked players (players with upper master levels) showed a higher black stone rate compared with lower-ranked players (players with lower master levels). We can speculate the mechanism using extant models for coin tossing probability, such as deterministic (Diaconis et al., 2007; Ferrie and Combes, 2014) and quantum models (Adler, 2014; Albrecht and Phillips, 2014), as a starting point. Both models may not be adequate in providing a plausible explanation for the unequal black stone rate, as this rate was significantly correlated to player’s ranking, but did not converge into a certain value, i.e., 0.51 in the case of deterministic model or 0.5 in the quantum model. Instead, it was likely that the setting and decision steps for stone selection were influenced by both players’ intentions, either subconsciously or consciously according to their preference for black stone, which could be supported by the previous studies on the significant first-move advantage for winning in Go (Kim and Kim, 1995; Kim and Jeong, 2005; Kim, 2007). In this regard, it cannot be completely ruled out that subtle interactions between players by potential unintended verbal or non-verbal cues (e.g., visual) might have influenced the black stone rates. However, it is typical to see that two Go players including professionals remain silent during the stone selection process. Therefore, it is critical to compare the black stone rate between ‘with’- vs. ‘without’- visual cues from the opponent player in a laboratory experimental study (Oh et al., 2025). At present, there are insufficient data available regarding, e.g., the number of white and black stones in the setting, decision steps and possibly others. After collecting various additional data including the number of grabbed stones in the stone selection, we may be able to suggest a plausible hypothesis for the underlying mechanism in which the inequality was observed. Combinations such as experimental investigation of and documentation for the number of stones in stone selection matches could be effective approaches for elucidating the mechanism. In fact, our recent report possibly provides a plausible mechanism which may underlie the discrepancies observed in the present study (Oh et al., 2025). In the laboratory study, the cancelation of the ambient geomagnetic field was sufficient for the significant reductions in the black stone rates for both men and women under lower blood glucose levels, in the absence of visual or auditory cues from the opponents. Moreover, precise analyses of the stone selection data including the number of grabbed white and black stone(s), revealed that geomagnetic sensing affected the binary decision-making subconsciously when the ordinary five senses were not effective. In this regard, *in situ* documentation of the number of grabbed stones in stone selection of professional or amateur Go games and the meal conditions of the Go players, may be helpful to comparatively investigate the underlying mechanism in the present and forthcoming studies.

From a game theory perspective, our findings may not be fully pertinent to the traditional assumption of convergence to mixed-strategy Nash equilibrium in sequential binary choice games (Nash, 1951). The presented data in the Figures 2, 4 are not from a *genuine* time series analysis. However, the results, especially Figure 2C raised the possibility of a deviation from the equilibrium with equal probabilities in the stone selections. The significant differences in stone selection patterns across ranking levels suggest that less experience, less habitual attitude or intuition toward stone selection might enable players to better recognize and exploit subtle strategic opportunities that seem to be a purely random choice situation. This aligns with recent findings in behavioral game theory in which expertise can lead to systematic deviations from theoretical equilibrium predictions (Camerer and Ho, 2015; Gauriot et al., 2023). The inverse relationship between professional career length and black stone selection success poses an interesting puzzle for strategic analysis. One possible explanation is that relatively newer professionals might be more adept at reading subtle behavioral cues or might approach the selection process with fewer preconceived notions about optimal strategies. Given the professional Go players could have experienced enough in the stone selection throughout their careers including as amateur Go players, the higher black stone rate observed in the relatively shorter professional career might be attributed to the relatively less experience as professionals rather than the young age of players *per se*. Note that the analyzed data on black stone rate are based on the length of their professional career, which may be correlated with their age, although we could not assess their age separately from the length of career, due to the limited personal information. In case of the amateur Go players in the *in situ* stone selection games, relatively longer experiences in Go games might produce favorable outcomes to higher master level (Dan) players, possibly through better reading non-verbal cues or experience-based intuition. Note that this scenario may be plausible due to the following reasons: (1) The higher Dan and the longer Go experiences were largely proportional to the ages of the amateur players, while their ages were in a relatively narrow range compared to that of professionals, (2) There are relatively short stone selection experience among the amateur players, compared to the professional players, which was not sufficient to nullify the effect of experience in the amateur groups. A possible reconciliation for the seemingly contradictory scenarios regarding “experience effect” above is that emerging professional players with relatively short professional career may be more motivated and focused on assembling the best alternative from the outset of the matches. While traditional game theory predicts that rational players should converge to mixed-strategy Nash equilibrium with equal probabilities in such binary choice situations (Nash, 1951), our findings suggest that experience and intuition may systematically influence strategic choices in the stone selections to result in unequal outcomes.

In testing the effect of gender on the black stone selection rate, only a portion of the dataset ― approximately 66% of the professional and 92% of the amateur matches ― was available for analysis in the early stage of the study, as gender information for certain players had not been recorded at the institutes. The marginal effects of gender did not substantially alter the main finding of the study, namely that the black stone selection rate of higher-ranked players was significantly higher than that of lower-ranked players, although these effects should be interpreted with caution due to the limitations of the available data. Noticeably, it is generally known that Go schools or academies, including the institutions where the stone selection games were taken place, do not teach or train skills/strategies for the stone selection. This information was verified through our personal interviews with the institutional representatives or teachers during our visits for the *in situ* games. Any of the possible skills or strategies may depend on Go players’ intentional or subconscious personal preferences toward black stone (Kim and Kim, 1995; Kim and Jeong, 2005; Kim, 2007). These suggest that strategic innovation even in seemingly simple binary choice situations might benefit somehow from fresh perspectives unencumbered by traditional approaches. In a view of game theory implications, the observed patterns in stone selection deviate from conventional game theory predictions in several ways. First, the remarkable correlations between ranking and selection outcomes suggest that some players may be able to gain strategic advantages over theoretically fair binary choices. Second, the sequential nature of selection process appears to create information asymmetries that skilled players can exploit, despite the theoretical prediction of strategy-proof mechanisms in such settings (Chen et al., 2023). Indeed, the results from the null simulation analysis support that the probability of higher-ranked players for black stones cannot be attributed to random variation, but rather represents a genuine strategic pattern. It appears that players in both professional and amateur matches engage in strategic behaviors to increase their chances of securing black. Interestingly, these findings align with the results of our recently published laboratory stone selection experiments, which demonstrated that both players attempted to increase their probability of obtaining black in a magnetic sense-dependent manner (Oh et al., 2025). The findings in the present study may contribute to the growing literature of subconscious effects on strategic interactions, suggesting that even in seemingly simple binary choice situations, experience, age, and/or intuition can contribute to systematic advantages (Lee et al., 2014; Lin and Hu, 2024).

In a retrospect, occasionally encountered apparent discrepancies between theoretical and empirical probabilities have usually been ignored, assuming that a discrepancy can be explained by the law of large numbers (Bernoulli, 1744) or deterministic mechanisms (Diaconis et al., 2007; Clark and Westerberg, 2009; Ferrie and Combes, 2014). From a broad perspective, unequal actual probability as identified in the present study may possibly appear in other binary choices (e.g., coin toss in football games). In this case, expecting rewards may motivate players to make a preferred decision for one side of the coin or the other. However, it is seemingly slightly different between the stone selection in Go game and coin-toss in the view of property of the decision-making. The stone selection can be regarded as a game rather than a simple event like a coin-toss, where the two players can have a command of their strategies intentionally but not randomly. Although there is a subtle difference in settings, they are similarly arranged on two steps for selection. The first step of tossing a coin or grabbing stones would be unintentional or subconscious, however, the second step would be more likely decision making as “head or tail” or “even or odd.” Therefore, we compared those two binary choices in our study. Nevertheless, within a limitation, the unequal actual probability has implications for our understanding of strategic decision-making in various contexts, including financial markets, e.g., stock markets, where binary choices are commonly under the uncertainties. The present study may provide insights of understanding the discrepancies between theoretical and empirical probability and raise the possible mechanism for sensory or strategic decision-making, which will help us understand the interaction modality among humans and between human and environment interface.

## Data Availability

The original contributions presented in the study are included in the article/Supplementary material, further inquiries can be directed to the corresponding authors.
