# Supplementary material for: Discrepancies between empirical and theoretical probability in human binary choices within the game of Go
Source: Front Psychol. 2026 Apr 30;17:1594220. doi: 10.3389/fpsyg.2026.1594220 (PMC13171542; doi:10.3389/fpsyg.2026.1594220)
Supplement: Supplementary file 1 [file Data_Sheet_1.pdf]

Record form (Stone selection game)

* Date : . . Hr : Min : * Location (room) :

* Player 1 (A) : Gender : Dan: Player 2 (B) : Gender : Dan: Game : Set :

Trial	Process		Result
	Step 1 (number of white stones)	Step 2 (number of black stones)	The player gets black stone
(Example 1)	A 17	B 1	B
(Example 2)	B 14	A 2	A
(Example 3)	A 15	B 2	A
1	A	B	
2	B	A	
3	A	B	
4	B	A	
5	A	B	
6	B	A	
7	A	B	
8	B	A	
9	A	B	
10	B	A	
11	A	B	
12	B	A	
13	A	B	
14	B	A	
15	A	B	
16	B	A	
17	A	B	
18	B	A	
19	A	B	
20	B	A	

Black stone rate (Set)
A: % (/20)
B: % (/20)

Black stone rate (Game)
A: % (/40)
B: % (/40)
