# Supplementary material for: Discrepancies between empirical and theoretical probability in human binary choices within the game of Go
Source: Front Psychol. 2026 Apr 30;17:1594220. doi: 10.3389/fpsyg.2026.1594220 (PMC13171542; doi:10.3389/fpsyg.2026.1594220)
Supplement: Supplementary file 7 [file Table_1.pdf]

Table S1. Professional Go matches data used for statistical analyses.

Year	Number of matches			Number of matches by compensation points [a]				
	Final round [b]	Preliminary round	Total	5.5	6.5	7.5	8	0 [c]
2000	520	2480	3000	749	2039	5	14	193
2001	533	2514	3047	177	2782	11	2	75
2002	775	2704	3479	299	2988	50	0	142
2003	687	2227	2914	1	2855	58	0	0
2004	711	2223	2934	1	2880	33	20	0
2005	925	3008	3933	0	3875	56	2	0
2006	1210	2455	3665	7	3624	34	0	0
2007	1158	3063	4221	1	4209	11	0	0
2008	987	2511	3498	3	3458	13	24	0
2009	811	2692	3503	0	3473	26	4	0
2010	1101	3046	4147	0	4124	23	0	0
2011	1157	3114	4271	0	4227	44	0	0
2012	1946	3573	5519	0	5366	126	27	0
2013	1505	3296	4801	0	4404	395	2	0
2014	1574	3829	5403	0	4281	1122	0	0
2015	1749	3449	5198	0	3836	1362	0	0
2016	1874	3011	4885	0	2935	1939	11	0
2017	1989	3323	5312	0	3638	1674	0	0
Total	21212	52518	73730	1238	64994	6982	106	410

The number of final-round matches analyzed for the 311 Go players was 21,212, as described in the Methods section. [a] The points added to the score of a player who played with white stones as a compensation for playing second in the game (Kim and Jeong, 2005; Kim, 2007). [b] Final rounds comprised final matches in championships or special tournaments. [c] Zero points were given in master level-up matches only.
