# Supplementary material for: Discrepancies between empirical and theoretical probability in human binary choices within the game of Go
Source: Front Psychol. 2026 Apr 30;17:1594220. doi: 10.3389/fpsyg.2026.1594220 (PMC13171542; doi:10.3389/fpsyg.2026.1594220)
Supplement: Supplementary file 8 [file Table_2.pdf]

Table S2. Re-analyzed data by the restricted likelihood ratio test using the same raw data analyzed in Figs. 2B and 4A.

Panel	n	Inclusion of event[a]	Log-likelihood	RLRT statistic	P-value
Fig. 2B (Professional)	13 923	Yes	− 8418.07	455.79	≈ 0
		No	− 8645.97		
Fig. 4A (Amateur)	5 580	Yes	− 3861.80	0.90	0.17
		No	− 3862.25		

[a] Official tournaments for professional and classrooms or training rooms for amateur.
