# Supplementary material for: Discrepancies between empirical and theoretical probability in human binary choices within the game of Go
Source: Front Psychol. 2026 Apr 30;17:1594220. doi: 10.3389/fpsyg.2026.1594220 (PMC13171542; doi:10.3389/fpsyg.2026.1594220)
Supplement: Supplementary file 9 [file Table_3.pdf]

Table S3. Re-analyzed data by the BT-GLMM *without* gender covariate using the same raw data analyzed in Figs. 2B and 4A.

Panel	*n*	Variable	Estimate	SE	t-Statistic	*P*-value	Deviance
Fig. 2D (Fig. 2B)	13 923	Intercept	− 0.2261	0.0386	− 5.8514	5.0×10^{-9}	− 0.3018, − 0.1504
		Δrank	0.0122	0.0003	36.7460	3.2×10^{-282}	0.0115, 0.0128
Fig. 4B (Fig. 4A)	5 580	Intercept	− 0.0356	0.0273	− 1.3031	0.1926	− 0.0892, 0.0180
		Δdan	0.0198	0.0072	2.7382	0.0062	0.0056, 0.0340
