# Supplementary material for: Discrepancies between empirical and theoretical probability in human binary choices within the game of Go
Source: Front Psychol. 2026 Apr 30;17:1594220. doi: 10.3389/fpsyg.2026.1594220 (PMC13171542; doi:10.3389/fpsyg.2026.1594220)
Supplement: Supplementary file 10 [file Table_4.pdf]

Table S4. Re-analyzed data by the BT-GLMM *with* gender covariate using the same raw data analyzed in Figs. 2B and 4A.

n	Variable	Estimate	SE	t-Statistic	*P*-value	Deviance
13 923 (Fig. 2B)	Intercept	− 0.2258	0.0386	− 5.8444	5.2×10^{-9}	− 0.3015, − 0.1501
	Δrank	0.0121	0.0003	36.9760	4.2×10^{-271}	0.0115, 0.0128
	Δgender	− 0.0934	0.0772	− 1.2093	0.2266	− 0.2448, 0.0580
5 120 (Fig. 4A)	Intercept	− 0.0267	0.0296	− 0.8997	0.3683	− 0.0847, 0.0314
	Δdan	0.0177	0.0078	2.2627	0.0237	0.0024, 0.0330
	Δgender	0.0452	0.0562	0.8039	0.4215	− 0.0650, 0.1553
