# Supplementary material for: Discrepancies between empirical and theoretical probability in human binary choices within the game of Go
Source: Front Psychol. 2026 Apr 30;17:1594220. doi: 10.3389/fpsyg.2026.1594220 (PMC13171542; doi:10.3389/fpsyg.2026.1594220)
Supplement: Supplementary file 11 [file Table_5.pdf]

Table S5. Re-analyzed data by the null simulation analysis using the same raw data analyzed in Figs. 2B and 4A.

Panel	*n*	Random variable[a]	β_1_real[b]	mean (β_1_sim[c])	SD	*P*-value
Fig. 4D (Fig. 2B)	13 923	Player 1	0.0122	− 0.000015	0.000235	≈ 0
		Player 2	0.0122	0.000005	0.000244	≈ 0
Fig. 4E (Fig. 4A)	5 580	Player 1	0.0198	− 0.000102	0.007220	0.0060
		Player 2	0.0198	0.000186	0.007269	0.0065

[a] The player whose real stone selections were replaced by random binary variables; [b] The β_1 from the BT–GLMM with the same raw data used in the corresponding figure; [c] The β_1 from the BT–GLMM with the simulated data for the corresponding figure; Replicates, 2 000.
